# ROR2 is epigenetically inactivated in the early stages of colorectal neoplasia and is associated with proliferation and migration

**DOI:** 10.1186/s12885-016-2576-7

**Published:** 2016-07-20

**Authors:** Sean S. Q. Ma, Sameer Srivastava, Estelle Llamosas, Nicholas J. Hawkins, Luke B. Hesson, Robyn L. Ward, Caroline E. Ford

**Affiliations:** Metastasis Research Group, Adult Cancer Program, School of Women’s and Children’s Health, Lowy Cancer Research Centre, UNSW Australia, Sydney, NSW 2052 Australia; Colorectal Cancer Group, Adult Cancer Program, Lowy Cancer Research Centre, UNSW Australia, Sydney, NSW 2052 Australia; Department of Biotechnology, Motilal Nehru National Institute of Technology Allahabad, Uttar Pradesh, 211004 India; Mayne Medical School, University of Queensland, 288 Herston Road, Herston, Brisbane St Lucia, Qld 4072 Australia

**Keywords:** Colorectal cancer, ROR2, Epigenetic silencing, Hypermethylation, Wnt

## Abstract

**Background:**

Colorectal cancer (CRC) is closely linked to Wnt signalling, with 94 % of cases exhibiting a Wnt related mutation. ROR2 is a receptor tyrosine kinase that is thought to repress β-catenin dependent Wnt signalling. Our study aims to determine if *ROR2* is epigenetically silenced in CRC and determine if in vitro silencing of *ROR2* potentiates Wnt signalling, and alters the proliferative, migratory or invasive potential of cells.

**Methods:**

*ROR2* expression was examined in CRC cell lines and patient adenomas using qRT-PCR, while COBRA and bisulphite sequencing was used to analyse *ROR2* promoter methylation. 258 patient primary tumour samples from publicly available databases were also examined for *ROR2* expression and methylation. In addition, the functional effects of *ROR2* modulation were investigated in HCT116 cells following *ROR2* siRNA knockdown and in RKO and SW620 cells following ectopic *ROR2* expression.

**Results:**

Reduced *ROR2* expression was found to correlate with *ROR2* promoter hypermethylation in colorectal cancer cell lines, carcinomas and adenomas. *ROR2* expression was downregulated in 76.7 % (23/30) of CRC cell lines with increasing *ROR2* promoter hypermethylation correlating with progressively lower expression. Analysis of 239 primary tumour samples from a publicly available cohort also found a significant correlation between reduced *ROR2* expression and increased promoter methylation. Methylation analysis of 88 adenomas and 47 normal mucosa samples found greater percentage of adenoma samples to be methylated. Additional analysis also revealed that adenoma samples with reduced *ROR2* expression also possessed *ROR2* promoter hypermethylation. *ROR2* knockdown in the CRC cell line HCT116 significantly decreased expression of the β-catenin independent Wnt targets genes *JNK* and *NFATC1*, increased cellular proliferation and migration but decreased invasion. When *ROR2* was ectopically expressed in RKO and SW620 cells, there was no significant change to either cellular proliferation or migration.

**Conclusion:**

*ROR2* is frequently epigenetically inactivated by promoter hypermethylation in the early stages of colorectal neoplasia and this may contribute to colorectal cancer progression by increasing cellular proliferation and migration.

**Electronic supplementary material:**

The online version of this article (doi:10.1186/s12885-016-2576-7) contains supplementary material, which is available to authorized users.

## Background

Colorectal cancer (CRC) is the third most common cancer worldwide with an estimated 1 million cases each year contributing to over 608,000 deaths [1–3]. CRCs develop from benign intraepithelial neoplasms known as adenomas, which progress to cancer after an accumulation of mutations [4, 5]. The Wnt signalling pathway is frequently altered in CRC with ~94 % of cases possessing a mutation in a Wnt pathway gene [6]. One of the early precipitating events for colorectal adenoma development is mutation of the *APC* gene, an important tumour suppressor and regulator of β-catenin dependent Wnt signals [5, 7, 8]. APC along with AXIN and GSK3β are responsible for degradation of cytosolic β-catenin and loss of APC leads to β-catenin accumulation, Wnt pathway hyperactivation and increased cellular proliferation and migration [8–15].

In contrast, the β-catenin independent Wnt pathway affects planar cell polarity (PCP), cell adhesion and motility and is not reliant on β-catenin levels [16–20]. The receptor tyrosine kinase-like orphan receptor 2 (ROR2) is a receptor tyrosine kinase which binds with WNT5A to activate the β-catenin independent Wnt pathway [21–23]. In addition to activating β-catenin independent Wnt/JNK signalling, ROR2 and WNT5A interaction has been shown to antagonise downstream targets of β-catenin dependent Wnt; specifically inhibition of *AXIN2* expression and the TCF/LEF transcription factors [16, 20, 23–26]. Consistent with its reported antagonism of β-catenin dependent Wnt signals, a 2010 study found *ROR2* to be silenced in colorectal cancer, resulting in increased cellular proliferation [27]. However, other reports in colorectal cancer, melanoma and osteosarcoma have found elevated *ROR2* expression in tumours compared to normal tissue [28–32]. These conflicting reports have raised questions as to the role ROR2 plays in cancer and presents the possibility that the downstream effects of ROR2 are dependent on other Wnt genes and the cellular context of the cancer itself [33–35].

In this study, we investigated whether *ROR2* expression is altered in colorectal cancers and adenomas. We also assessed the effects of altered *ROR2* expression on β-catenin dependent Wnt signalling, proliferation, migration and invasion properties in colorectal cancer cells.

## Results

### ROR2 is epigenetically silenced by promoter hypermethylation in colorectal cancer cell lines

**Fig. 1:**
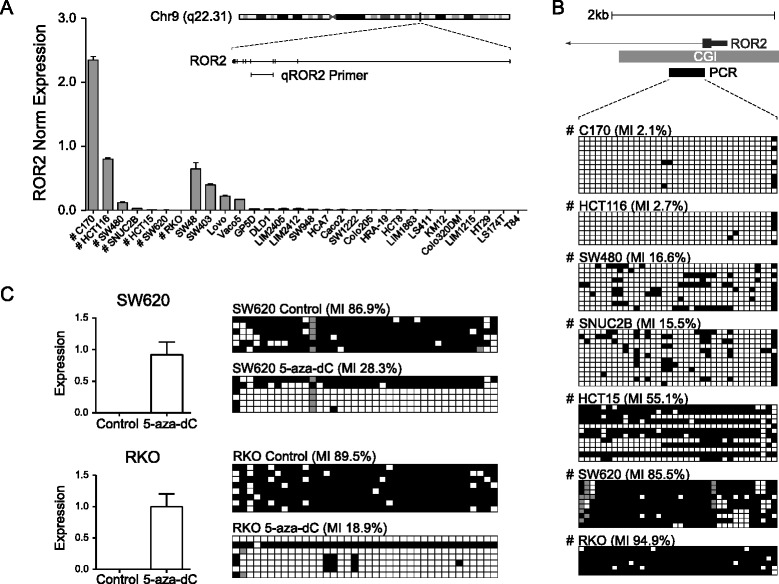
*ROR2* expression loss in colorectal cancer cell lines caused by promoter hypermethylation. **a** qRT-PCR of 30 different colorectal cancer cell lines showing *ROR2* expression normalised against 3 housekeeping genes. Insert shows the relative position of *ROR2* qRT-PCR primers relative to *ROR2* gene. **b** Bisulphite sequencing of 7 colorectal cancer cell lines (C170, HCT116, SW480, SNUC2B, HCT15, SW620, RKO) showing increased methylation index (MI) of *ROR2* promoter correlating with decreased levels of *ROR2* mRNA expression. Black squares represent methylated CpG dinucleotides. White squares represent unmethylated CpG dinucleotides. Grey squares represent CpG dinucleotide with an inconclusive finding. Gene map of *ROR2* indicates the region of the *ROR2* CpG island analysed in bisulphite sequencing. **c** qRT-PCR of RKO and SW620 cells after 5-aza-2-deoxycytidine (5-aza-dC) treatment compared with control cells (*n =* 3). *ROR2* expression was normalised against 3 housekeeping genes. Corresponding bisulphite sequencing reveals loss of *ROR2* promoter methylation and decreased methylation index (MI) resulting from 5-aza-dC treatment

Quantitative reverse transcriptase polymerase chain reaction (qRT-PCR) showed 23 out of 30 CRC cell lines lacked expression of *ROR2* at the mRNA level (Fig. 1a). Methylation analysis using combined bisulphite restriction analysis (COBRA) showed 25 out of the 30 cell lines had methylation in the *ROR2* promoter (Additional file 1).

Bisulphite sequencing revealed that C170 and HCT116 cell lines, which had the highest levels of *ROR2* expression, had little to no methylation across the examined promoter molecules. SW480, SNUC2B and HCT15 cell lines which have low levels of *ROR2* expression were revealed to have higher levels of methylation across their promoter molecules. The cell lines RKO and SW620 with no detectable levels of *ROR2* expression were found to have heavy promoter methylation (Fig. 1b).

Treatment of 2 methylated cell lines (SW620 and RKO) with the DNA methyltransferase inhibitor 5-aza-2′-deoxycytidine (5-aza-dC) resulted in *ROR2* promoter demethylation and restoration of *ROR2* expression (Fig. 1c).

### Epigenetic inactivation of ROR2 is an early event in colorectal neoplasia

**Fig. 2:**
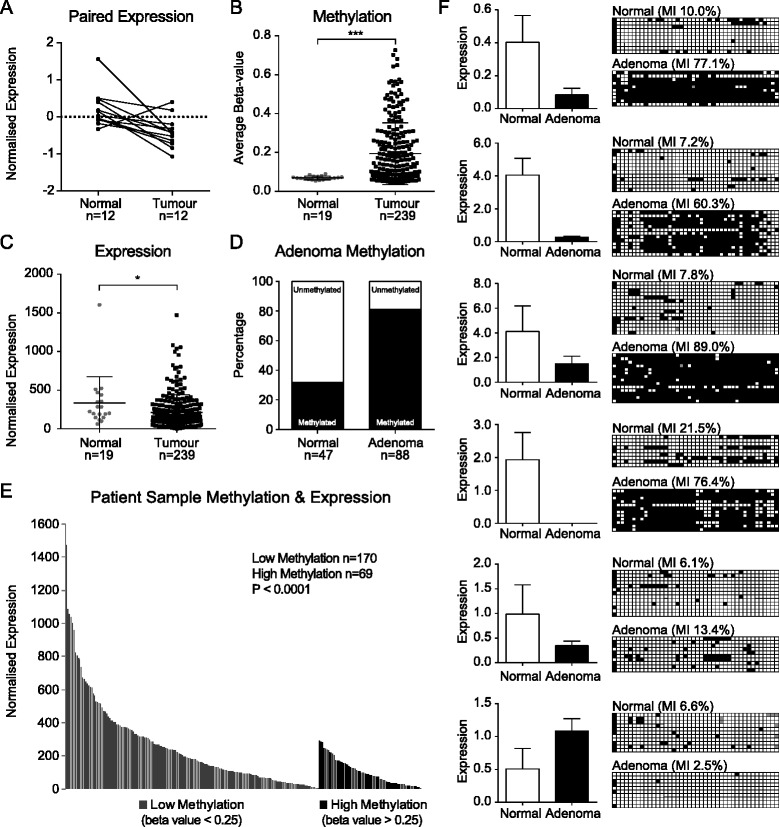
*ROR2* promoter hypermethylation and silencing in adenomas and patient tumour samples. **a** Matching normal and tumour samples from TCGA database showing differences in *ROR2* expression as assessed using Agilent microarray (*n =* 12) (*P <* 0.01). **b** *ROR2* methylation comparison in entire cohort of tumour and normal samples from TCGA database as assessed using Illumina Infinium (HumanMethylation450) arrays (*n =* 258) (*P <* 0.001). Methylation values were obtained by averaging the beta values of the methylation probes that fell within the *ROR2* CpG island. **c** Average normalised *ROR2* expression in entire cohort of tumour and normal samples from TCGA database as assessed using Illumina RNA-Seq (*n =* 258) (*P <* 0.05). **d** Methylation percentages in colorectal adenomas and normal samples as analysed using COBRA assays (*n =* 47 & *n =* 88 respectively). **e** Comparison of *ROR2* expression to methylation in colorectal tumour samples from TCGA database (*n =* 239) (*P <* 0.0001). Samples with average beta values <0.25 were categorised as low methylation whilst samples with average beta values >0.25 were categorised as high methylation. The results shown here are based upon data generated by the TCGA Research Network: http://cancergenome.nih.gov/. **f** qRT-PCR of 6 patient adenoma samples with matching normal tissue showing differences in *ROR2* expression. Expression was normalised against 3 housekeeping genes. Bisulphite sequencing revealing a corresponding change in *ROR2* promoter methylation between samples of patient adenomas and adjacent normal tissue

To determine if *ROR2* expression was also reduced in primary tumour samples, we examined publicly available data from The Cancer Genome Atlas (TCGA). Data from 12 paired CRC patient samples showed that on average, 11 of the patient primary tumours had a twofold decrease in *ROR2* expression compared to the normal mucosa samples (*P* < 0.01) (Fig. 2a).

*ROR2* methylation was examined in a larger cohort of 239 CRCs and 19 normal mucosa samples and significantly greater methylation was found in the CRCs (*P* < 0.001) (Fig. 2b). Examination of the RNA-Seq data within the cohort also found significantly lower *ROR2* expression (*P* < 0.05) in the CRCs compared to the normal (Fig. 2c). A direct comparison of methylation and expression in the colorectal tumour samples of the cohort revealed that samples with high methylation (beta values > 0.25) had significantly lower *ROR2* expression (*P* < 0.0001) (Fig. 2e, Additional file 2). This analysis of publicly available data reveals that loss of *ROR2* expression is present in CRCs as well as cell lines and that hypermethylation of the *ROR2* CpG island (CGI) is the cause.

To determine whether hypermethylation of the *ROR2* promoter was an early or late event in colorectal neoplasia, we compared the number of methylated samples in 88 non-invasive adenomas to 47 normal mucosa specimens. COBRA assays revealed methylation in 80.7 % of adenomas while only 15.5 % of the normal mucosa showed signs of methylation (Fig. 2d). *ROR2* expression and methylation were examined in 6 adenoma samples chosen for their absence of submucosal infiltration and non-serrated histological profile (Additional file 3). qRT-PCR revealed 5 of the 6 adenomas had reduced *ROR2* expression compared to matching normal mucosa samples. Bisulphite sequencing showed that 4 of those adenomas were hypermethylated across the *ROR2* CGI promoter (Fig. 2f).

### In vitro silencing of ROR2 in colorectal cancer increases proliferation and migration and decreases invasion

**Fig. 3:**
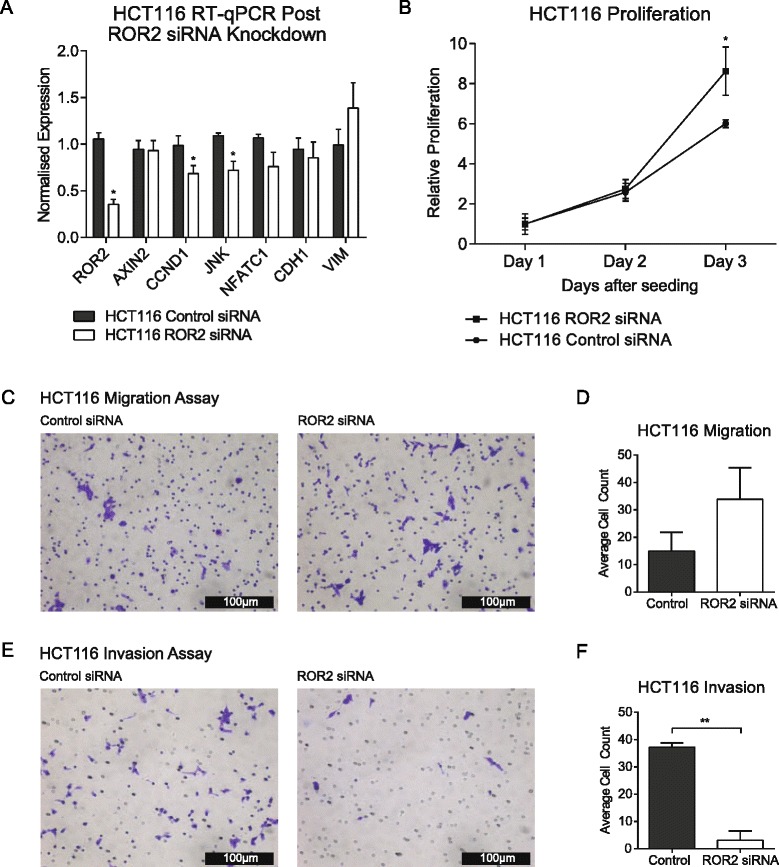
Increased proliferative, metastatic and invasive potential following *ROR2* knockdown in HCT116 cells. **a** qRT-PCR of Wnt & EMT associated genes in HCT116 cell lines after *ROR2* siRNA knockdown. All expression results normalised against 3 housekeeping genes (*n =* 3) (*P <* 0.05). **b** CCK-8 proliferation assay of HCT116 cells with and without *ROR2* siRNA knockdown (*n =* 3) (*P <* 0.01). **c** Images of transwell migration assay of HCT116 cells with and without *ROR2* siRNA knockdown at 10× magnification. **d** Average cell count comparison between HCT116 cells with and without *ROR2* siRNA knockdown. Average count taken from 4 independent image fields at 20× magnification (*n =* 3). **e** Images of transwell invasion assay of HCT116 cells with and without *ROR2* siRNA knockdown at 10× magnification. **f** Average cell count comparison between HCT116 cells with and without *ROR2* siRNA knockdown. Average count taken from 4 independent image fields at 20× magnification (*n =* 3) (*P <* 0.01)

To explore the effects of loss of *ROR2* expression on Wnt signalling, we utilised siRNA knockdown of *ROR2* mRNA and assessed the expression of the β-catenin independent Wnt genes *JNK* and *NFATC1*, the β-catenin dependent genes *AXIN2* and *CCND1* and the epithelial-mesenchymal transition markers *VIM* and *CDH1*. Silencing *ROR2* in the HCT116 cell line was associated with a 34 % reduction in *JNK* (*P* < 0.01) and 29 % reduction in *NFATC1* (*P* < 0.05) and 31 % reduction in *CCND1* (*P* < 0.05). *ROR2* knockdown did not result in significant changes to *VIM*, *AXIN2* and *CDH1* expression levels (Fig. 3a). To assess the effects of *ROR2* loss on cell behaviour, we next assessed proliferation, migration and invasion kinetics.

*ROR2* silencing significantly increased the proliferation at HCT116 cells (*P* < 0.05) (Fig. 3b). Transwell migration assays suggested a marginal increase in cellular migration, though this did not reach statistical significance (*P =* 0.056) (Fig. 3c). However, the ability of cells to invade through an extracellular matrix was decreased following *ROR2* silencing (*P <* 0.01) (Fig. 3d). These data show *ROR2* loss in HCT116 cells results in changes in the expression of a specific subset of Wnt signalling genes, increases proliferation and migration but decreases invasion.

### Ectopic expression of ROR2 in RKO and SW620 cell lines did not significantly alter cellular proliferation, migration and invasion

**Fig. 4:**
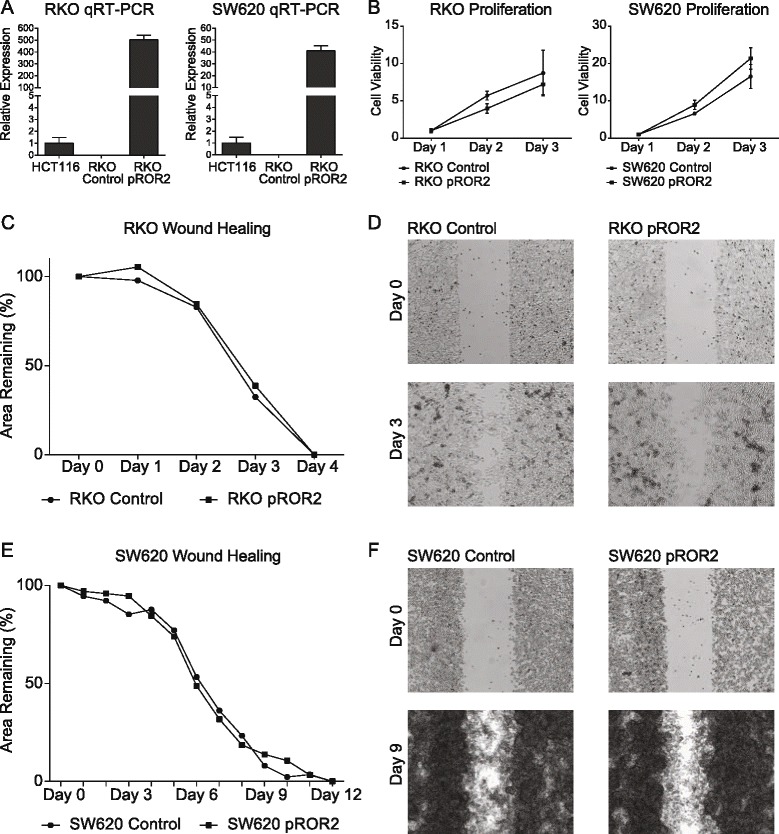
Functional consequences of ectopic *ROR2* expression in RKO and SW620 cell lines. **a** *ROR2* qRT-PCR of RKO and SW620 cell lines with ectopic *ROR2* expression (pROR2 transfection) and control (pFLAG-CMV-4™ transfection) relative to expression in HCT116 cell lines. All expression results normalised against 3 housekeeping genes (*n =* 1). **b** CCK-8 proliferation assay of RKO and SW620 cells with and without ectopic *ROR2* expression (*n =* 3). **c** Wound healing assay comparing percentage area of wound covered by RKO cells with and without ectopic *ROR2* expression over a 4 day period (*n =* 1). **d** Images of RKO cells in wound healing assay on day 0 and day 3 comparing cells with and without ectopic *ROR2* expression. **e** Wound healing assay comparing percentage area of wound covered by SW620 cells with and without ectopic *ROR2* expression over a 12 day period (*n =* 1). **f** Images of SW620 cells in wound healing assay on day 0 and day 9 comparing cells with and without ectopic *ROR2* expression

*ROR2* was ectopically expressed in RKO and SW620 cells using a *ROR2* pFLAG plasmid (pROR2) with re-expression of the receptor confirmed using qRT-PCR (Fig. 4a). Following ectopic *ROR2* expression in RKO and SW620 cells, there was no significant change to cellular proliferation (Fig. 4b). When cellular migration was examined in RKO and SW620 cells using wound healing assays, there was no significant change detected between the rate of wound closure between cells with and without ectopic *ROR2* expression (Fig. 4c-f), indicating that although *ROR2* knockdown may resulted in functional changes to CRC cell lines, the same may not be true with ectopic *ROR2* expression.

## Discussion

Although *ROR2* is not normally expressed in mature adult cells, evidence from prior studies indicate that it is present in the colon epithelium as well as in parathyroid, testicular and uterine tissue [27, 36]. Previous publications examining *ROR2* in CRC found both upregulation and downregulation of the receptor in CRC [27, 28]. Both publications used qRT-PCR to document *ROR2* expression in 20 matching tumour and normal samples yet report different findings. The reasons for the conflicting results in these 2 publications remain unclear although differences in study cohort and methodology may explain this discrepancy.

In our study, analysis using qRT-PCR found *ROR2* expression loss in the majority of both CRC cell lines (*n =* 23) and colorectal adenoma (*n =* 6) samples. In addition, analysis of 258 patient samples from the publicly available TCGA database found a significant decrease of *ROR2* expression in primary tumour samples compared to the normal mucosa, providing strong evidence that *ROR2* is downregulated in CRC.

Our study also uses COBRA and bisulphite sequencing to show for the first time that not only is promoter hypermethylation present in the majority of CRC cell lines but it is also present in early colorectal adenomas. Along with the methylation, there was also a corresponding loss of *ROR2* expression in the adenoma samples, leading us to hypothesise that the observed downregulation was caused by epigenetic silencing through promoter hypermethylation. This was supported by our analysis of both CRC cell lines and primary tumours samples from the publicly available TCGA database as well as data from the previous publication from Lara et al. [27]. Our cell line experimentation also supported this hypothesis as *ROR2* expression was restored in RKO and SW620 cells following demethylation using the DNA methyltransferase inhibitor 5-aza-2′deoxycytidine. These findings of *ROR2* expression loss and promoter hypermethylation are particularly important as they have been conducted on not only cell lines but also on clinical samples from both adenomas and primary tumours. Together, the clinical data along with the analysis and experimentation of cell lines provides strong evidence that epigenetic silencing of ROR2 through promoter hypermethylation occurs early in colorectal carcinogenesis.

Although we have shown *ROR2* to be epigenetically silenced in the majority of CRC cases, the exact molecular outcomes of this loss in the colon epithelium remains unclear. Knockdown experiments confirm that *ROR2* expression loss results in a subsequent decrease of the downstream β-catenin independent Wnt genes *JNK* and *NFATC1*. Although previous studies have shown *ROR2* loss resulted in increased expression of the β-catenin dependent Wnt target AXIN2 [23], we did not observe this in our in vitro cell line model. Our examination of the β-catenin dependent Wnt target *AXIN2* and *CCND1* not only revealed no apparent increase but *CCND1* expression levels were instead found to be significantly decreased. A likely explanation for this difference in findings may be the differences in the biological models used, as the previous publication which reported increased *AXIN2* expression following *ROR2* silencing used in vivo mouse models incorporating the tumour microenvironment [24]. It is possible that in our experiments on an immortal cancer cell line, the cellular and genetic context was significantly different and that the loss of *ROR2* resulted in the activation of different signalling pathways. This is supported by recent publications which show *ROR2* and other Wnt associated genes to be capable of activating both the β-catenin dependent and β-catenin independent halves of the Wnt signalling pathway [20, 24, 33, 34, 37]. *ROR2* has been shown to interact with different co-receptors [38, 39] and ligands [40] as well being the target of phosphorylation by different intracellular proteins [25, 41]. As the exact signalling consequences of these *ROR2* interactions are as yet uncertain, it is possible that *ROR2* downregulation resulted in different signalling cascades in in vivo mice and in immortal cancer cells.

Although there was no observed upregulation in the β-catenin dependent Wnt target genes following *ROR2* knockdown as reported in the literature [23, 24], our in vitro assays on HCT116 cells still revealed an increase in proliferation and migration. There was a significant increase to cellular proliferation following *ROR2* knockdown while the observed increase to migration was close to significance with a P value of only 0.056. The effect of *ROR2* knockdown on cellular invasion was also investigated in HCT116 cells, with the results revealing a decrease in cellular invasion. These results are consistent with our findings that *ROR2* was initially lost in precancerous adenomas which possess no invasive properties. Analysis of gene expression also found no changes to the key EMT-related genes *CDH1* and *VIM* following *ROR2* knockdown, suggesting that invasion capacity in CRC only occurs later during disease progression. Our combined functional analysis indicates that *ROR2* downregulation may cause increased proliferation and migration in early non-invasive adenomas, resulting in a more metastatic phenotype. The lack of observed increase in β-catenin dependent Wnt target genes indicate that these changes were not influenced by the inhibition of β-catenin dependent Wnt signals. It is possible that *ROR2* loss affected both arms of the Wnt signalling pathways as had been previously reported in breast and renal cancer, resulting in the observed phenotypic changes [20, 33–35]. Another possibility is that the interaction between Wnt signalling and another signalling pathway resulted in unexpected circumstances [42–44]. It is evident that *ROR2* plays a much more complex role in CRC and the Wnt signalling pathway than previously thought. Further investigations examining the direct interactions *ROR2* has with Wnt and EMT associated genes through techniques such as DNA microarrays or RNA-seq would help reveal the exact mechanism in which *ROR2* affects cellular proliferation and migration in the context of CRC progression.

As we had found loss of *ROR2* function to increase cellular proliferation and migration, we hypothesised that re-expression of the receptor may have the opposite effect. However, when *ROR2* was ectopically expressed in RKO and SW620 cells, there was no significant change observed in cellular proliferation and migration. This may have been because the level of *ROR2* expression generated by plasmid transfection was significantly higher than that of normal *ROR2* expression levels. This could have adversely affected Wnt signalling as certain pathways are sensitive to the ratios of receptors and ligands [45, 46].

It is also possible that the RKO cell line was not functionally affected by ectopic *ROR2* expression because it did not originate from a CRC caused by aberrant Wnt signalling. Although ~94 % of CRC cases possess a mutation in a Wnt pathway gene with *APC* being the gene most predominantly mutated [6], not all CRC cases arise from a dysfunctional Wnt signalling pathway. A significant proportion of CRC cases result from other causes such as mutations and methylation in mismatch repair (MMR) genes [47, 48]. RKO cells do not have a mutant *APC* gene but they do have methylated MMR genes as well as possessing the CpG island methylator phenotype [49]. This suggests that RKO cells originally became carcinogenic through methylation and loss of function in MMR genes rather than through aberrant Wnt signalling.

In SW620 cells, the absence of any change in proliferation and migration following ectopic ROR2 expression may have been because the cell line originated from a secondary tumour site. SW620 and SW480 cells originated from the same patient with SW620 cells obtained from a lymph node metastasis while SW480 were from the primary tumour [50]. Having already metastasised to a secondary site, SW620 cells would possess a markedly different genetic composition than that of a primary tumour and may be resistant to any functional effects resulting from restoration of expression in an early gene target such as *ROR2*.

This is a potential issue for all cell line models as they are cancer cells that are different to the colorectal adenomas in which we believe *ROR2* methylation and expression loss first occurs. To truly determine if early *ROR2* loss is involved in CRC progression in adenomas, a biological model which more closely resembles colorectal adenomas would be needed. Future research could possibly investigate functional effects of *ROR2* loss in colorectal adenomas grown in in vitro organoids [51]. Another possibility would be to use an inducible mouse knockout model that targeted *ROR2* in the colon. Using a mouse strain that had a high prevalence for adenomas such as the APC heterozygous 57BL/6 J-ApcMin/J mouse line, would allow for the determination of whether or not early ROR2 loss potentiates adenoma growth and development.

## Conclusion

Our study has found that *ROR2* promoter hypermethylation and subsequent expression loss is an early event in CRC progression that first occurs in non-invasive adenomas. *ROR2* expression was found to be downregulated in the majority of CRC cases, with subsequent in vitro experimentation indicating that the silencing of the receptor may facilitate increased cellular proliferation and migration. Although it was hypothesised that hyperactivation of the β-catenin dependent Wnt signals was the cause, decreases in both β-catenin dependent and independent genes following *ROR2* knockdown suggested that the effects of *ROR2* modulation are context dependent and that the observed effects on proliferation and migration may be influence by interactions with pathways other than β-catenin dependent Wnt [35, 43, 52, 53]. Future research investigating the interaction of ROR2 with various Wnt and EMT associated proteins would help elucidate the exact mechanism in which ROR2 affects cellular proliferation and migration. Examination of *ROR2* loss in a more adenoma like biological model instead of in cancer cell lines would also aide in determining if the silencing of the receptor promoted CRC progression.

## Methods

### Cell lines

All colorectal cancer cells were obtained from ATCC (American Type Culture Collection, Manassas, VA, USA). HCT116 cells were cultured in McCoy’s media (Life Technologies, Rockville, MD) supplemented with 10 % foetal bovine serum, 1× glutamine (200 mM) and penicillin/streptomycin (10 units/ml). RKO cells were cultured in RPMI media (Life Technologies, Rockville, MD) supplemented with 10 % foetal bovine serum, 1× glutamine (200 mM) and penicillin/streptomycin (10 units/ml). SW620 cells were cultured in DMEM (Life Technologies, Rockville, MD) supplemented with 10 % foetal bovine serum, 1× glutamine (200 mM) and penicillin/streptomycin (10 units/ml). Cells were grown in incubators with humidified atmosphere of 5 % CO_2_ at 37 °C. Cells were tested on a monthly basis to ensure there was no mycoplasma contamination.

### ROR2 pFLAG plasmid construction

A ROR2 pFLAG plasmid (pROR2) was constructed by isolating the ROR2 cDNA transcript from the Addgene ROR2 plasmid using Primer 1 (CTGATATCGATGGCCCGGGGCTCGGCGCTCCCGC) and Primer 2 (TCCTCTAGATCAAGCTTCCAG CTGGACTTGG). The resulting PCR fragment then underwent restriction enzyme digestion with both EcoRV and XbaI. The DNA was then subcloned into the pFLAG-CMV™-4 plasmid containing an N-terminal epitope tag following a similar restriction enzyme digest.

### ROR2 siRNA Knockdown

Cells were seeded at 1 × 10^6^ cells into 60 mm plates (Nunc™, Thermo Fisher Scientific, Rockford, IL USA) and allowed to adhere over a 6 h period. Cells were then serum starved for 18 h before being transfected with either 60 pmoles of *ROR2* siRNA or scrambled control siRNA (Life Technologies, Rockville, MD). siRNA were premixed in 250 μl of serum free McCoy’s media (Life Technologies, Rockville, MD). siRNA mixture was then combined with 6 μl of Lipofectamine® 2000 (Life Technologies, Rockville, MD) premixed in 250 μl of serum free McCoy’s media before addition to cells. After transfection, cells were incubated at 5 % CO_2_ at 37 °C before being used in subsequent experimentation.

### Ectopic ROR2 expression

Cells were seeded at 1 × 10^6^ cells into 60 mm plates (Nunc™, Thermo Fisher Scientific, Rockford, IL USA) and allowed to adhere over a 6 h period. Cells were then serum starved for 18 h before being transfected with either 1.4 μg of empty pFLAG-CMV™-4 plasmid or 1.4 g of pmoles of *ROR2* pFLAG plasmid. Plasmid solutions were premixed in 250 μl of serum free RPMI media (Life Technologies, Rockville, MD) for RKO cells and DMEM (Life Technologies, Rockville, MD) for SW620 cells. The plasmid solutions were then combined with 6 μl of Lipofectamine® 2000 (Life Technologies, Rockville, MD) premixed in 250 μl of the appropriate serum free media before addition to cells. After transfection, cells were incubated at 5 % CO_2_ at 37 °C before being used in subsequent experimentation.

### Quantitative real time PCR

Cell samples underwent cell lysis using 2-mercaptoethanol and RNA extraction was carried out using the RNeasy Extraction Kit (Qiagen 74106). 1 μg of RNA was quantified and treated with RNase-free DNase (Life Technologies 18068–015). The DNase treated RNA was used for cDNA synthesis using Quantitect cDNA synthesis kit (Qiagen 205313) with appropriate negative controls. The primer sequence used for *ROR2* qRT-PCR was designed to amplify a region which included all known transcript variants of *ROR2* (Forward 5′-GTCCAACGCACAGCCCAAATC-3′ & Reverse 5′-CCGGTTGCCAATGAAGCGTG-3′). qRT-PCR was performed using SYBR® Mastermix Reagent (Qiagen 204056) and the M × 5000p Thermal Cycler. Each sample was run in triplicate and the experiment was run for 40 cycles. *ROR2* results and those of Wnt & EMT related genes (AXIN2, CCND1, JNK, NFATC1, CDH1, VIM) were normalised against 3 house-keeping genes (SDHA, RPL13A, HSP90AB1). Primer sequences for additional genes can be found in Additional file 4. *ROR2* knockdown qRT-PCR experiments were repeated in triplicate and statistical significance was evaluated using unpaired *t*-test.

### Combined bisulphite restriction analysis (COBRA) Assay

DNA was extracted from samples before undergoing bisulphite treatment using Ez DNA Methylation™ – Gold Kit (Zymo Research, Australia). The *ROR2* promoter region was amplified using *ROR2* COBRA semi-nested primers which covered a 436 bp region of the 1958 bp *ROR2* CpG island where MBD-Seq data indicated the greatest level of coverage. (Forward 5′-GGGTTAYGTTTATTTTAGGATTTTGTTAGGT-3′ & Forward nested 5′-GTYGTGTGTTTTTGAAGGAGGAAGATT-3′ & Reverse 5′-CTCTCAATATCCCRAACTTCAAATAAAATCTAA-3′). The PCR product was digested with TaqI restriction enzyme (Fermentas) before undergoing gel electrophoresis in a 1.5 % agarose gel. Resulting bands were visualised under UV light.

### Bisulphite sequencing

DNA was extracted from samples before undergoing bisulphite treatment using Ez DNA Methylation™ – Gold Kit (Zymo Research, Australia). *ROR2* COBRA semi-nested primers were used to amplify the *ROR2* CpG island region. The resulting PCR product was then ligated into pCR™2.1-TOPO® plasmid (Life Technologies, Rockville, MD) before being transformed into chemically competent DH5α™ *E. coli* bacteria. The bacteria were utilised to clone the PCR product before being plated onto LB agar plates for blue white selection. Bacteria which contained pCR™2.1-TOPO® plasmid with *ROR2* PCR inserts were sequenced using BigDye® (Life Technologies, Rockville, MD) with *ROR2* Reverse and Forward nested primers before undergoing Sanger sequencing (Ramaciotti Centre, UNSW Australia).

### 5-aza-2-deoxycytidine treatment

Cells were seeded at 1 × 10^6^ cells into 60 mm plates (Nunc™, Thermo Fisher Scientific, Rockford, IL USA) and allowed to adhere over a 24 h period. Cells were subsequently treated to 2.5 μM concentrations of 5-aza-2-deoxycytidine (Sigma A3656). Treatment was repeated every 24 h over a 72 h period. Control cells were treated with the vehicle control of acetic acid instead of 5-aza-2-deoxycytidine.

### Data analysis of TCGA cohort

Normalised *ROR2* expression and methylation data of tumour and matched normal tissue were obtained from The Cancer Genome Atlas (http://cancergenome.nih.gov/) and analysed by Agilent microarrays and Illumina HiSeq 2000 RNA Sequencing. Methylation values were analysed using Illumina Infinium (HumanMethylation450) arrays and the beta-value average was obtained from methylation probes that fell within the 1958 bp *ROR2* CpG island. Statistical significance of matched patient tumour and normal samples were carried out using paired *t*-test. Statistical significance of expression and methylation comparison of the entire cohort was evaluated using unpaired *t*-test. Statistical significance of expression in low and high methylation samples was evaluated using unpaired *t*-test. The results shown in these analyses are in whole or part based upon data generated by the TCGA Research Network; http://cancergenome.nih.gov/.

### Patient samples

Forty-seven normal and 88 adenoma samples were collected from patients at Westmead Hospital using endoscopic mucosal resection (Ethics committee approval number 2008/6/4.6 and 11194, Sydney West Area Health Service Human Research and Ethics Committee) [54]. A further six fresh colorectal adenomas and paired adjacent normal mucosa samples were taken from surgical resection specimens from 3 males and 3 females at St Vincent’s Hospital, Sydney (Ethics committee approval number H00/022 and 00113) [55]. Informed consent was obtained from all patients participating in the study. The adenomas obtained showed no evidence of invasive malignancy (Additional file 3).

### Proliferation assay

Twenty four h after *ROR2* siRNA transfection, *ROR2* knockdown and control HCT116 cells were lifted using 1× 0.5 % Trypsin EDTA and seeded into a clear 96-well well plate (Nunc™, Thermo Fisher Scientific, Rockford, IL USA) at 1 × 10^4^ cells/well. Cells were allowed to adhere for 2 h before 3 wells of *ROR2* knockdown cells and 3 wells of control cells were treated with 10 μl of CCK-8 reagent (Dojindo Molecular Technologies, Inc. Rockville, MD) before the plate was wrapped in foil. 10 μl of CCK-8 reagent was also added to 3 media only wells to act as control blank readings. 2 h after addition of CCK-8 reagent, the treated wells were read on Spectramax 190 plate reader at 450 nm absorbance using the media only wells as blank readings. CCK-8 reagent was applied to additional triplicate wells at 24 & 48 h after the initial seeding and their 450 nm absorbance was read to determine changes in cellular proliferation. All subsequent readings for each siRNA treatment was normalised against the initial reading 2 h after seeding. The experiment was repeated in triplicate and statistical significance was evaluated using 2 way ANOVA.

### Migration assay

Seven hundred μl of media supplemented with 20 % foetal bovine serum was added to the lower chamber of transwell migration plates while 200 μl of media supplemented with 1 % foetal bovine serum was added to the insert (Corning Incorporated – Life Sciences, One Becton Circle Durham, NC 27712 USA). 24 h after *ROR2* siRNA transfection, knockdown and control HCT116 cells were lifted using 1× 0.5 % Trypsin EDTA and resuspended in 1 % FBS media to a concentration of 7 × 10^5^ cells/ml. 100 μl of cell solution was added to the inserts. The plates were incubated for 48 h at 37 °C before the inserts were removed and washed twice in PBS. Cells were then fixed with 100 % methanol for 20 min before again being washed twice in PBS. Inserts were then stained with 1 % crystal violet for 30 min before being washed twice in PBS. Non-migrated cells on the upper surface of inserts were removed using cotton swabs. The transwell membrane was then excised and mounted onto a glass slide with mounting medium (Dako CS70330-2). 4 independent field counts at 20× magnification using ImageQuant TL Software were used to assess cell numbers. The experiment was repeated in triplicate and statistical analysis was evaluated using unpaired *t*-test.

### Invasion assay

Transwell invasion plates with pre-coated matrigel were first rehydrated using warm serum free media for 2 h at 37 °C. Media was then removed and 750 μl of media supplemented with 20 % foetal bovine serum was added to the lower chamber while 100 μl of serum free media was added to the insert (Corning Incorporated–Life Sciences, One Becton Circle Durham, NC 27712 USA). 24 h after *ROR2* siRNA transfection, knockdown and control HCT116 cells were lifted using 1× 0.5 % Trypsin EDTA and resuspended in 1 % FBS media to a concentration of 7 × 10^5^ cells/ml. 200 μl of cell solution was added to the inserts. The plates were incubated for 48 h at 37 °C before the inserts were removed and washed twice in PBS. Cells were then fixed with 100 % methanol for 20 min before again being washed twice in PBS. Inserts were then stained with 1 % crystal violet for 30 min before being washed twice in PBS. Non-migrated cells on the upper surface of inserts were removed using cotton swabs. The transwell membrane was then excised and mounted onto a glass slide with mounting medium (Dako CS70330-2). 4 independent field counts at 20× magnification using ImageQuant TL Software were used to assess cell numbers. The experiment was repeated in triplicate and statistical analysis was evaluated using unpaired *t*-test.

## Abbreviations

5-aza-dC, 5-aza-2′-deoxycytidine; ATCC, American Type Culture Collection; CGI, CpG island; COBRA, combined bisulphite restriction analysis; CRC, colorectal Cancer; MI, methylation index; MMR genes, Mismatch repair genes; PCP, planar cell polarity; qRT-PCR, quantitative reverse transcriptase polymerase chain reaction; ROR2, receptor tyrosine kinase-like orphan receptor 2; TCGA, The Cancer Genome Atlas

## Additional files

Additional file 1: COBRA of CRC cell lines showing methylation in majority of samples. COBRA assays on 31 colorectal cancer cell lines reveals that 26 of the cell lines possessed some level of *ROR2* methylation. (EPS 3931 kb) Additional file 2:
*ROR2* methylation and expression correlation. Analysis of correlation between *ROR2* methylation and expression in 239 primary tumour samples from TCGA dataset. (EPS 568 kb) Additional file 3: Table of qRT-PCR Primer Sequences. Histopathological information on adenoma samples and matching normal mucosa. Age ranges of patients 57–79. Samples listed in order from top to bottom in Fig 2F. (TXT 552 bytes) Additional file 4: Table of qRT-PCR Primer Sequences. List of qRT-PCR primer sequences used in experimentation. (TXT 726 bytes)
